# Deeply branching *Bacillota* species exhibit atypical Gram-negative staining

**DOI:** 10.1128/spectrum.00732-24

**Published:** 2024-08-20

**Authors:** Jessica K. Choi, Saroj Poudel, Nathan Yee, Jennifer L. Goff

**Affiliations:** 1 Ecology and Evolutionary Biology Department, University of Michigan, Ann Arbor, Michigan, USA; 2 Department of Marine and Coastal Sciences, Rutgers, New Brunswick, New Jersey, USA; 3 Department of Earth and Planetary Sciences, Rutgers, New Brunswick, New Jersey, USA; 4 Department of Environmental Sciences, Rutgers, New Brunswick, New Jersey, USA; 5 Department of Chemistry, SUNY College of Environmental Science and Forestry, Syracuse, New York, USA; South China Sea Institute of Oceanology Chinese Academy of Sciences, Guangzhou, Guangdong, China

**Keywords:** cell wall, evolution, genomics, Gram-positive bacteria, Gram-negative bacteria

## Abstract

The Gram staining method differentiates bacteria based on their cell envelope structure, with the monoderm and diderm cell envelope types traditionally being synonymous with Gram-positive and Gram-negative stain results, respectively. Monoderms have a single phospholipid membrane surrounded by a thick layer of peptidoglycan, while diderms have a lipopolysaccharide outer membrane exterior to a thin peptidoglycan layer. The *Bacillota* (formerly *Firmicutes*) phylum has members with both cell wall types, and recent phylogenetic analyses have shown that monoderm *Bacillota* evolved from diderm ancestors on multiple occasions. Here, we compiled Gram staining and ultrastructural data for *Bacillota* species with complete genomes to further investigate the evolution of Gram-positive and Gram-negative cell wall types. The results indicate that many deeply branching lineages at the root of *Bacillota* phylum stain Gram-negative but do not harbor genes for outer membrane protein or lipopolysaccharide biosynthesis. Phylogenetic reconstructions suggest that several deeply branching *Bacillota* species have retained a thin peptidoglycan layer in their cell walls, which was inherited from a diderm ancestor. Taxa with this atypical Gram-negative-staining cell wall structure include the thermophilic anaerobe *Symbiobacterium thermophilum* and members of the *Desulfotomaculia, Syntrophamonadia, Desulfitobacteriia, Thermosediminibacteria,* and *Thermoanaerobacteria*. Using Gram-staining results as a proxy for cell wall thickness, our analysis indicates that several independent peptidoglycan thickening events may have occurred in the evolution of the Gram-positive cell envelope.

**IMPORTANCE:**

In this study, we examined the evolution of bacterial cell envelopes, specifically focusing on Gram-positive and Gram-negative cell wall types in the *Bacillota* phylum. Our results indicate that certain bacteria can stain Gram-negative despite having a monoderm cell wall structure, thus challenging the conventional interpretation of Gram-staining results. Our observations also question the assumption that Gram-negative staining is always indicative of a diderm structure. These findings have broader implications for understanding how and when cell walls thicken during the evolution of bacterial cell envelopes.

## OBSERVATION

Over billions of years, bacteria have undergone changes in their cell envelopes giving rise to the Gram-positive and Gram-negative cell wall types (1). The Gram staining method is used to differentiate bacteria based on their cell envelope structure (2) with diderm cell envelopes traditionally being synonymous with Gram-negative stain results. The chemistry of the staining procedure reflects the thickness of the peptidoglycan layer of the cell wall (3). The primary staining agent, crystal violet (CV), freely enters both monoderm and diderm cells. The application of Gram’s iodine solution results in a precipitated CV-iodide complex within the cytoplasm. The thick monoderm cell wall retains the CV-iodide precipitates during the organic solvent decolorizing step, resulting in the deep purple coloring that typifies Gram-positive bacteria. In contrast, diderms have an outer membrane (OM) located exterior to a thinner peptidoglycan cell wall (4). Decolorization disrupts diderm OM, and the thinner peptidoglycan layer is unable to retain the CV-iodide complex, resulting in the loss of the CV stain. Counterstaining results in the final red/pink appearance typical of Gram-negative bacteria. The canonical OM in Gram-negative diderm bacteria is an asymmetric lipid bilayer, with a lipopolysaccharide outer membrane (LPS-OM) and outer membrane proteins (OMPs).

Because the *Bacillota* (previously known as the Firmicutes) members encompass both monoderm and diderm cell envelope types, there is significant interest in understanding the evolutionary history of this phylum to discern when and how the cell envelope transition occurred. Systematic and taxonomic studies from the past two decades have established that this phylum also contains Gram-negative-staining diderm members in the classes *Negativicutes* (5, 6), *Halanaerobiia* (7, 8), and *Limnochordia* (9). Like the canonical Gram-negative bacteria (e.g*.,* the *Pseudomonadota*, formerly the *Proteobacteria*), the cell walls of *Negativicutes* and *Halanaerobiia* are comprised of an LPS-containing OM exterior to a thin peptidoglycan layer. The genomes of *Negativicutes* and *Halanaerobiia* encode complete sets of genes for the biosynthesis of an LPS-OM, including genes for the synthesis of the lipid A and lipopolysaccharide transport system for exporting LPS across the periplasm to the outer leaflet of the OM (1, 10, 11). Phylogenetic analyses have shown that Gram-positive monoderms evolved from Gram-negative diderm ancestors through multiple LPS-OM loss events (1, 10–13). Recently, it was suggested that the thickening of the peptidoglycan layer in Gram-positive species may have been automatic following OM loss in monoderms (14); however, the mechanistic details of this transition remain unclear. We propose that the Gram-stain result of a bacterium can be used as a proxy for the thickness of the peptidoglycan cell wall. We hypothesized that among the monoderm *Bacillota*, representatives of intermediary cell wall types exist that lack an LPS-OM but still have a thin peptidoglycan wall, resulting in a Gram-negative stain.

We analyzed a total of 366 representative complete *Bacillota* genomes and identified numerous atypical Gram-negative species with multi-layered cell envelopes that lack LPS-OM biosynthesis genes (Table 1). All analysis methods are described in detail in the Supplemental materials file. Compilation of previously published data indicates that these organisms stain Gram-negative and that their cell envelopes contain one or more outer surface layers that enclose a thin peptidoglycan layer. The genomes of these atypical Gram-negative strains do not contain genes for lipid A biosynthesis (*lpxABCD*) or LPS transport (*lptA*), demonstrating that the observed outer layers are not composed of lipopolysaccharide. Their genomes also lack genes for the outer membrane protein OmpH and the OMP assembly protein BamA, further confirming that these outer layers are not diderm OMs. In certain cases, the outer layer is a protein S-layer (15–18). Atypical Gram-negative species with this unusual cell wall structure include the anaerobic thermophiles *Symbiobacterium thermophilum*, *Ammonifex degensii*, *Syntrophothermus lipocalidus*, *Thermoanaerobacterium aotearoense, Thermoanaerobacter kivui*, *Thermoanaerobacter tengcongensis, Pseudoclostridium thermosuccinogenes*, and *Novibacillus thermophilus*. Others in this group include the anaerobes *Syntrophomonas wolfei*, *Dehalobacter restrictus, Desulfosporosinus acidiphilus, Flavonifractor plautii*, *Lachnoclostridium phytofermentans*, *Clostridium scatologenes*, and *Christensenella minuta*.

**TABLE 1:** Ultrastructural data for atypical Gram-negative *Bacillota* that lack LPS genes

Species^*a*^	Gram stain	Reported ultrastructure description	LpxABCD^*b*^	LptA	OmpH	BamA
*Halanaerobium praevalens DSM 2228*	Negative	Outer-wall membranous layer	●	●	●	●
*Selenomonas sputigena ATCC 35185*	Negative	Convoluted outer double layer	●	●	●	●
*Bacillus subtilis*	Positive	Monoderm and thick cell wall	○	○	○	○
*Symbiobacterium thermophilum IAM 14863*	Negative	Three-layered cell wall structure	○	○	○	○
*Ammonifex degensii KC4*	Negative	Surface layer exterior to peptidoglycan layer	○	○	○	○
*Syntrophothermus lipocalidus DSM 12680*	Negative	Outer layer exterior to the peptidoglycan layer	○	○	○	○
*Syntrophomonas wolfei Goettingen G311*	Negative	Outer membrane	○	○	○	○
*Dehalobacter restrictus*	Negative	Proteinaceous outer S-layer	○	○	○	○
*Desulfosporosinus acidiphilus SJ4*	Negative	Multilayered cell wall	○	○	○	○
*Thermoanaerobacter kivui*	Negative	Crystalline outer surface layer	○	○	○	○
*Thermoanaerobacter tengcongensis*	Negative	Outer layer exterior to thin inner layer	○	○	○	○
*Thermoanaerobacterium aotearoense*	Negative	Cell surface covered with S-layer	○	○	○	○
*Flavonifractor plautii*	Negative	Multilayered cell wall	○	○	○	○
*Pseudoclostridium thermosuccinogenes*	Negative	Multilayered cell wall; S-layer	○	○	○	○
*Lachnoclostridium phytofermentans ISDg*	Negative	Multilayered cell wall	○	○	○	○
*Christensenella minuta*	Negative	Gram-negative cell wall structure	○	○	○	○
*Clostridium scatologenes*	Negative	Multilayered cell wall	○	○	○	○
*Novibacillus thermophilus*	Negative	Gram-negative type of cell wall	○	○	○	○
*Thermanaeromonas toyohensis*	Negative	Monoderm	○	○	○	○
*Thermacetogenium phaeum DSM 12270*	Negative	Monoderm	○	○	○	○
*Thermosediminibacter oceani*	Negative	Monoderm	○	○	○	○
*Caldicellulosiruptor owensensis*	Negative	Monoderm	○	○	○	○
*Syntrophobotulus glycolicus DSM 8271*	Negative	Monoderm	○	○	○	○
*Oscillibacter valericigenes Sjm18-20*	Negative	Monoderm	○	○	○	○
*Mageeibacillus indolicus UPII9-5*	Negative	Monoderm	○	○	○	○
*Cellulosilyticum lentocellum DSM 5427*	Negative	Monoderm	○	○	○	○
*Heliorestis convoluta*	Negative	Monoderm	○	○	○	○
*Heliobacterium modesticaldum Ice1*	Negative	Monoderm	○	○	○	○

^*a*^ Data sources for each species are reported in Table S1.

^*b*^ Accession numbers and amino acid sequences of query proteins are shown in Table S2; a total of 3,854 complete Bacillota genomes were analyzed, and results are shown in Table S2; full white circle (○) indicate the absence of the gene, a full black circle (●) indicate the presence of the gene.

We also found numerous examples of monoderms that stain Gram-negative (Table 1). Our data compilation indicates that these organisms stain Gram-negative even though transmission electron microscopy images show a monoderm cell wall structure. None of these organisms have genes for LpxABCD, LptA, OmpH, and BamA, further supporting the absence of an LPS-OM. The Gram-negative stain results are likely due to a thin peptidoglycan layer in the cell wall [e.g., as described in reference (19)]. Examples of Gram-negative-staining monoderms are *Thermanaeromonas toyohensis, Thermacetogenium phaeum*, *Thermosediminibacter oceani, Caldicellulosiruptor owensensis, Syntrophobotulus glycolicus*, *Oscillibacter valericigenes*, *Cellulosilyticum lentocellum*, and *Mageeibacillus indolicus*. Gram-negative-staining monoderms also include the anaerobic phototrophs *Heliobacterium modesticaldum* and *Heliorestis convoluta*. In total, we found 45 Gram-negative *Bacillota* species that lack LPS-OM biosynthesis genes (Table S1) that we collectively describe as “atypical Gram-negative” bacteria. These atypical Gram-negative bacteria stain Gram-negative but lack a diderm cell envelope and/or lack LPS-OM biosynthesis genes.

Among our 366 representative genomes, we identified two with singleton *lpx* genes (Table S1): *lpxC* in *Desulfotomaculum acetoxidans* (20) and *lpxA* in *Cohnella abietis* (21). While an ultrastructure is not reported for either of these two species, both belong to *Bacillota* classes with monoderm representatives (*Desulfotomaculia* and *Bacilli*, respectively, Table S1). *D. acetoxidans* stains Gram-negative while *C. abietis* stains Gram-positive (Table S1). An expanded search of 3,855 *Bacillota* genomes (Table S2), found a partial *lpx* operon (*lpxACD*) in the Gram-positive *Paenibacillus crassostreae* (22) and an LpxC homolog in *Peptococcaceae* bacterium DCMF (23) (also referred to as *Candidatus Forminomas warabiya*), a member of the monoderm class *Dehalobacteriia* (Table S1). A phylogenetic analysis was performed to determine if these genes were re-acquired by horizontal gene transfer or if they represent “vestigial” genes remaining after the loss of the LPS-OM. A detailed analysis with methods and an expanded discussion of results is reported in the Supplemental materials. Briefly, the *lpxACD* genes of *P. crassostreae* and the *lpxA* gene of *C. abietis* appear to have been re-acquired by a single horizontal gene transfer from members of the *Terrabacteria* (Fig. S1 to S4). In contrast, the *lpxC* genes in *D. acetoxidans* and *Peptococcaceae* bacterium DCMF may represent genuine remnants of a paralogous l*pxC2* gene region found in some *Bacillota* (Fig. S5 and S6) that is distinct from the main LPS-OM biosynthesis gene cluster. However, as two out of four of these strains with *lpx* genes stain Gram-positive, we suggest that these isolated *lpx* genes are unlikely to contribute to the atypical Gram-negative phenotype. Based on the function of characterized *lpx* paralogues in other bacteria, these genes may instead have novel roles in virulence (24, 25), temperature adaptation (25), and other environmental stress responses (26).

**Fig 1:**
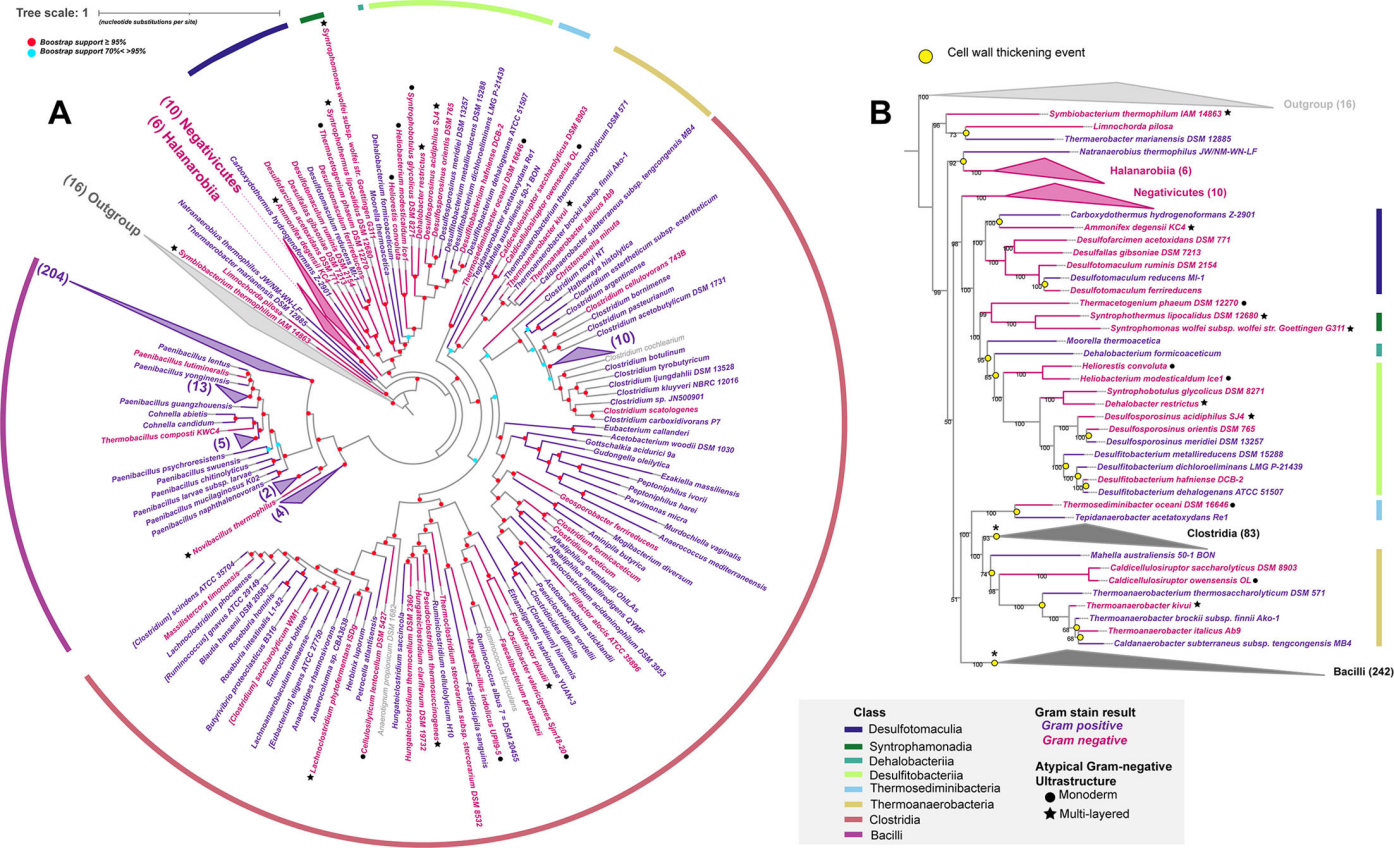
*Bacillota* ribosomal protein tree annotated with Gram stain phenotypes. The *Bacillota* maximum-likelihood phylogenetic tree was reconstructed using 55 conserved ribosomal proteins from representative genomes (*n* = 361, Table S3). The outgroup (*n* = 16) for the tree was taken from reference (10). (**A**) Expanded tree showing, where available, Gram-staining information indicated by the color of the branch/strain name (Gram-negative is pink and Gram-positive is purple). The color legend is also shown in the figure. Gram-positive and Gram-variable strains are both colored purple. *Bacillota* classes are shown on the outer circle. The outgroup clade (*n* = 16) is collapsed for visualization purposes. All other collapsed clades contain members that all stain Gram-negative or all stain Gram-positive. These were collapsed for simplicity. The number of members in each collapsed clade is indicated in parentheses. For ease of visualization, we have replaced bootstrap values with red (>=95% branch support) or blue (70%< >95% branch support) dots. The legend for these dots is also visible on the figure under the tree scale. (**B**) Tree with the *Bacilli* and *Clostridia* classes collapsed to highlight a subset of the cell wall thickening events (marked by a yellow circle). *Clostridia and *Bacilli* clades are collapsed but have evidence for multiple thickening events (see panel A) occurring within both classes. The legend at the bottom is for both panels. In both panels—excluding *Limnochorda pilosa, the Negativicutes*, and *Halanaerobiia*—all Gram-negative strains are what we describe as “atypical” Gram-negative *Bacillota* (i.e*.,* stain Gram-negative but lack LPS-OM biosynthesis genes, Table S1). Atypical strains with confirmed monoderm ultrastructure (Table 1) are indicated by black circles. Atypical strains with ultrastructure described as “multi-layered” (Table 1) are indicated by black stars.

To examine the phylogeny of the atypical Gram-negative *Bacillota*, we generated a concatenated ribosomal protein tree (Fig. 1A) overlaid with the Gram staining data. The ancestral state of the *Bacillota* is a Gram-negative diderm cell envelope (1, 10–13), and our phylogenetic reconstruction indicates that the atypical Gram-negative cell wall type was inherited from the diderm ancestor with a thin cell wall peptidoglycan layer. *S. thermophilum* IAM 14863 is the deepest branching atypical Gram-negative species and is closely related to the diderm lineages of the *Bacillota*. This organism has a thin peptidoglycan cell wall (27) but lacks LPS-OM biosynthesis genes, thus demonstrating that LPS-OM gene loss occurred without subsequent cell wall thickening. We also identified several deeply branching atypical Gram-negative lineages—members of the *Desulfotomaculia, Syntrophamonadia*, and *Desulfitobacteriia*—that are closely related to the *Negativicutes*, a class of diderm *Bacillota*. Like, *S. thermophilum*, these deeply branching atypical Gram-negative organisms lost their LPS-OM biosynthesis genes but did not undergo subsequent thickening of their cell walls. Two later-diverging classes, the *Thermosediminibacteria* and *Thermoanaerobacteria*, also have this thin peptidoglycan layer cell wall type while lacking LPS-OM biosynthesis genes. In total, among the deeper branching monoderm lineages (i.e*.,* the *Thermaerobacteria, Syntrophamonadia, Symbiobacteriia, Moorellia, Desulfotomaculia,* and *Dehalobacteriia*) 15 out of the 17 (88%) analyzed genomes stain Gram-negative. In contrast, the Gram-positive phenotype (i.e*.,* a thick peptidoglycan layer) is common among the later-branching *Clostridia* (63/84 analyzed *Clostridia* genomes stain Gram-positive) and *Bacilli* (233/242 analyzed *Bacilli* genomes stain Gram-positive). Interestingly, many of the atypical Gram-negative lineages across the *Bacillota* tree are thermophilic organisms (Table S5). Even among the *Bacilli*, two of the three (*N. thermophilus, Thermobacillus composti*) atypical Gram-negative strains are thermophilic. Prior work with the facultative thermophile *Bacillus coagulans* showed that growth at 55°C, compared to 37°C, resulted in lower teichoic acid content and reduced peptide cross-bridging in its peptidoglycan cell wall (28). This decreased peptide cross-bridging may have a role in limiting the thickness of the peptidoglycan cell wall in these thermophilic atypical Gram-negative lineages.

The phylogeny of Gram-positive and Gram-negative species indicate that several independent peptidoglycan thickening events have transpired in the evolutionary history of the *Bacillota* (Fig. 1B). These results suggest that peptidoglycan cell wall thickening did not happen automatically after OM loss in the evolution of monoderm cell envelopes. We hypothesize that the thickening of the peptidoglycan layer may have been driven primarily by changes in gene regulation in response to environmental cues (28). Future work should compare regulatory differences in cell wall formation between canonical Gram-positive monoderm *Bacillota* and atypical Gram-negative monoderm *Bacillota*. Additionally, *Thermaerobacter marianensis*—one of the earliest monoderms (Fig. 1)—stains weakly Gram-positive during the exponential phase of growth and Gram-negative during the stationary phase (29). The genome of this deeply branching *Bacillota* lacks the Lpx, Lpt, and OMP machinery required for synthesizing an LPS-OM. Because *T. marianensis* is one of the earliest monoderms to diverge from the ancestral *Bacillota* diderm, it could be a candidate representing the early evolution of the Gram-positive cell wall type.

## SUPPLEMENTAL MATERIAL

Supplemental materials spectrum.00732-24-s0001.docx Supplemental methods and figures.

Table S1 spectrum.00732-24-s0002.xlsx *Bacillota* ultrastructural and genomic features.

Table S2 spectrum.00732-24-s0003.xlsx Accession numbers of LPS-OM-related proteins found in the *Bacillota*.

Table S3 spectrum.00732-24-s0004.xlsx Representative genomes along with their identified ribosomal proteins.

Table S4 spectrum.00732-24-s0005.xlsx Accession numbers and amino acid sequences of query proteins used in this study.

Table S5 spectrum.00732-24-s0006.xlsx Complete structural, genomic, and growth temperature information for the atypical Gram-negative strains.

ASM does not own the copyrights to Supplemental Material that may be linked to, or accessed through, an article. The authors have granted ASM a non-exclusive, world-wide license to publish the Supplemental Material files. Please contact the corresponding author directly for reuse.
