# Supplementary material for: Deeply branching Bacillota species exhibit atypical Gram-negative staining
Source: Microbiol Spectr. 2024 Aug 20;12(10):e00732-24. doi: 10.1128/spectrum.00732-24 (PMC11448272; doi:10.1128/spectrum.00732-24)
Supplement: Supplemental materials — Supplemental methods and figures. [file spectrum.00732-24-s0001.docx]

**SUPPORTING INFORMATION FOR**

**Deeply branching *Bacillota* species exhibit atypical Gram-Negative staining**

^1^Jessica K. Choi, ^2^Saroj Poudel, ^3,4^Nathan Yee, ^5,*^Jennifer L. Goff

^1^Ecology and Evolutionary Biology Department, University of Michigan, Ann Arbor, MI, USA

^2^Department of Marine and Coastal Sciences, Rutgers-New Brunswick, NJ, USA

^3^Department of Earth and Planetary Sciences, Rutgers-New Brunswick, NJ, USA

^4^Department of Environmental Sciences, Rutgers-New Brunswick, NJ, USA

^5^Department of Chemistry, SUNY College of Environmental Science and Forestry, Syracuse, NY, USA

*Corresponding author:

(ORCID ID: 0000-0002-9089-9632)

Department of Chemistry

SUNY College of Environmental Science and Forestry

1 Forestry Dr.

Syracuse, NY, 13210, USA

Email: [jegoff@esf.edu](mailto:jegoff@esf.edu)

Phone: 315-470-6824

**SUPPLEMENTAL METHODS**

***Identification of LPS and OMP homologs.*** A database comprising 3855 complete genomes from the *Bacillota* phylum deposited in the National Center for Biotechnology Information (NCBI) database as of May 2020 was locally created. We searched these *Bacillota* genomes for homologs of LpxABCD, LptA, OmpH, and BamA. LpxABCD are involved in the biosynthesis of Kdo_2_-Lipid A (1). LptA is involved in lipopolysaccharide (LPS) transport across the periplasm (2). BamA is involved in the assembly and insertion of proteins into the outer membrane (OM) (3). OmpH is an OM protein (4). For this search, representatives of each protein from the *Bacillota* phylum were first curated from the literature (5, 6). For any remaining proteins where a representative could not be identified from the literature, corresponding sequences from *Escherichia coli* were first used as query sequences via NCBI’s protein Basic Local Alignment Search Tool (BLASTp) again the *Negativicutes* and *Halanaerobiia*. The best result (query coverage ≥ 60%, similarity ≥ 30%, and e-value <= 0) was chosen as the representative sequence for each protein **(Table S4).** In our *Bacillota* genomes, homologous sequences for each representative protein were identified using the *phmmer* program via the HMMER software package (v3.3.1) (7) The resulting protein sequences were scanned for protein domains against the Pfam database (8) with an e-value cutoff of 1x10^-5^.

***Ribosomal Tree.*** The NCBI identifies genomes suitable for use as representative strains. Consequently, we curated a set comprising all these representative genomes within the *Bacillota*, totaling 366, for the purpose of reconstructing a multilocus phylogenetic trees using conserved ribosomal proteins. Outgroups (n=16) for the tree were taken from Taib et al. (9). A total of 55 ribosomal proteins from *E. coli* were used as reference sequences (10). Homologs of these ribosomal proteins were identified and extracted from our representative genomes using the *phmmer* program as described above and SAMtools (11) **(Table S3).** A total of 45 ribosomal proteins were present in > 97% of the genomes. Extracted homologous ribosomal protein sequences were individually aligned with default settings using Clustal Omega (12) and concatenated in sequential order as shown in **Table S3**. Aligned blocks were used to reconstruct a maximum-likelihood phylogenetic tree using IQ-TREE (13). The Model Finder (14) implemented in IQ-TREE was used to find the best mixture model with the base substitution model Li Gascuel (LG) (15). The AIC (Akaike Information Criterion) and BIC (Bayesian Information Criterion) in the Model Finder found LG+I+I+R10 to be the best fit model, which was used to reconstruct the tree with 1000 ultrafast bootstrap iterations (15) The resulting tree was then visualized using iTol (16).

***Literature review for cell envelope structure data.*** To integrate morphological data with phylogeny and LPS and OM gene occurrences, we performed a literature search on the 364 representative *Bacillota* strains with completed genomes that are not members of the characterized diderm classes *Limnochordia, Halanaerobiia*, or *Negativicutes.* This list included 13 *Bacillota* classes generally thought to be monoderms (9). Two major pieces of structural information were extracted from the compiled literature: *(1)* Gram-stain results and *(2)* ultrastructural data (*i.e.,* transmission electron microscopy imagining) **(Table S1).** For the Gram-stain, we noted whether the organism stained Gram-positive or negative, where reported. For instances of Gram-variability, we scored these organisms as Gram-positive because it is common for monoderms that stain Gram-positive early in growth to stain Gram-negative as the cultures age due to the deterioration of the cell envelope (17). In total, we compiled at least partial structural data from 355 (~98%) of the representative strains, with coverage of all 13 *Bacillota* classes analyzed. Of these, 60 had both staining and ultrastructural data available.

***Lpx Trees.*** Phylogenetic analyses were carried out with the LpxA, LpxC, and LpxD homologs identified in monoderm *Bacillota* genomes. Lpx protein trees were constructed using the representative sequences reported in Antunes et al. (18). We added additional representatives of the GTDB clade “*Bacillota* G” as this clade includes the diderm class Limnochordia and close relatives as there were not represented in the Antunes et al. (18) analysis. To examine the evolution of these homologs, we constructed individual LpxA and LpxC trees as well as a concatenated LpxADC maximum likelihood tree. Sequence alignment was performed using Clustal Omega (12) implemented in Geneious Prime (v2023.1.2). Alignments were trimmed using the trimAl tool (v1.2) with the parameters *-automated1 -clustal* (19). Trimmed alignments were used to construct maximum-likelihood trees in IQ-TREE (v2.2.2.6) (13) running ModelFinder with parameters *-B 3000 -mset LG*. ModelFinder selected the following models: LpxA – LG+R6; LpxC – LG+I+R8; LpxD – LG+R5.  The trimmed LpxA, LpxC, and LpxD alignments were concatenated and used to construct a maximum-likelihood tree in IQ-TREE (v2.2.2.6) with parameters *-B 3000 -mset LG*. ModelFinder selected the LG+I+R7 model. The resulting trees were visualized in iToL (16).

**SUPPLEMENTAL TEXT ON PHYLOGENETIC ANALYSIS FOR LPX GENES**

For *P. crassostreae*, we constructed a concatenated LpxADC protein tree **(Fig. S1).** The majority of the diderm *Bacillota* LpxADC sequences form a single clade except for *L. pilosa* and three close, uncultivated relatives. The LpxADC sequences from these four strains form a sister clade alongside the closely related *Cyanobacteriota* and *Deinococcota.* However, the *P. crassostreae* LpxADC sequence does not cluster with any of the diderm *Bacillota* lineages. Instead, it forms a clade with the *Verrucomicrobiota* and *Chlamydiota.* While the *Bacillota*, along with the *Cyanobacteriota* and *Deinococcota*, are members of the larger clade *Terrabacteria*, the *Verrucomicrobiota* and *Chlamydiota* are more distantly related and members of the *Gracilicutes* (20). Additionally, this *P. crassostreae lpxACD* genomic region is distinct from the lipopolyssacharide outer membrane (LPS-OM) biosynthetic cluster “scar” region previously described by Taib et al. (9) in other monoderm *Bacillota* genomes. *P. crassostreae* has this “scar” region, with the genes *murA, spoIID, spoIIID, mreB, flgG, flgF,* and *fabZ* present **(Fig. S2).**

An expanded search for LPS-OM homologs in a larger database of monoderm *Bacillota* genomes also found an LpxA homolog in a close relative of *P. crassostreae*, *Cohnella abietis. C. abietis* stains Gram positive; however, no ultrastructural data are reported (21). In contrast to the *P. crassostreae lpxA,* this *lpxA* gene is not accompanied by any other LPS-OM biosynthesis genes at its genomic loci **(Fig S3A).** It is also separate from the LPS-OM “scar” region found elsewhere in the *C. abeitis* genome **(Fig. S3B).** We constructed an LpxA protein tree and found that the *C. abietis* LpxA clusters with the *P. crassostreae* LpxA but not the LpxAs of any diderm *Bacillota* **(Fig S4).** Thus, we propose that the *C. abietis lpxA* region is the result of the same horizontal gene transfer event as the *P. crassostreae lpxADC* region. While, ultrastructural data is not reported for *C. abietis* or *P. crassostreae*, both stain Gram positive (22, 23) and lack all other LPS-OM biosynthetic genes **(Table S2)—**suggesting that they do not have an OM.

We also observed LpxC homologs in two deeper branching monoderm *Bacillota* lineages: *Peptococcaceae bacterium* DCMF (24) and *Desulfofarcimen acetoxidans* (25). Strain DCMF is not in pure culture, so its cell envelope structural features are unknown. However, it does not have homologs to any of the other *lpx* genes or any other essential genes for the LPS-OM biosynthesis **(Table S2).** *D. acetoxidans* stains Gram-negative (25). While no ultrastructural data are reported for *D. acetoxidans,* *Desulfofarcimen intricatum* was shown to have a monoderm cell wall structure using the KOH lysis test (26). The *lpxC* genes in strain DCMF and *D. acetoxidans* are in a conserved genomic region, flanked by genes with only broad and non-specific annotations **(Fig. S5A).** Like the *P. crassostreae* and *C. abietis* Lpx homologs, these *lpxC* homologs are in a distinct genomic region from the LPS-OM biosynthetic cluster “scar” region **(Fig. S5B).**Interestingly, we found syntenic regions in several genomes of the closely related diderm *Negativicutes:* *Sporomusa acidovorans* DSM 3132, *Sporomusa silvacetica* DSM 10669, and *Sporomusa malonica* DSM 5090 **(Fig. S5A)**. These *lpxC2* regions are distinct from the main LPS-OM biosynthetic cluster. We speculate that they many have functions in responding to varying environmental stressors (27). Thus, we hypothesized that the *lpxC* observed in strain DCMG and *D. acetoxidans* may represent remnants of a paralogous *lpxC2* region in the ancestral diderm *Bacillota*. To test this hypothesis, we constructed an LpxC protein tree. *D. acetoxidans* and Strain DCMG LpxC sequences form a clade with the *Bacillota* Limnochordaceae bacterium strains Bu26 and Bu89; Firmicutes bacterium strain AS02xzSISU_7; and the closely related *Terrabacteria* in the *Dictyoglomota*, *Cyanobacteriota*, and *Deinococcota* **(Fig. S6).** From these data, we propose that the *lpxC* in these two strains are likely “vestigial” *lpxC2* paralogues rather than *lpxC1* genes re-acquired by horizontal gene transfer.

**SUPPLEMENTAL FIGURES**


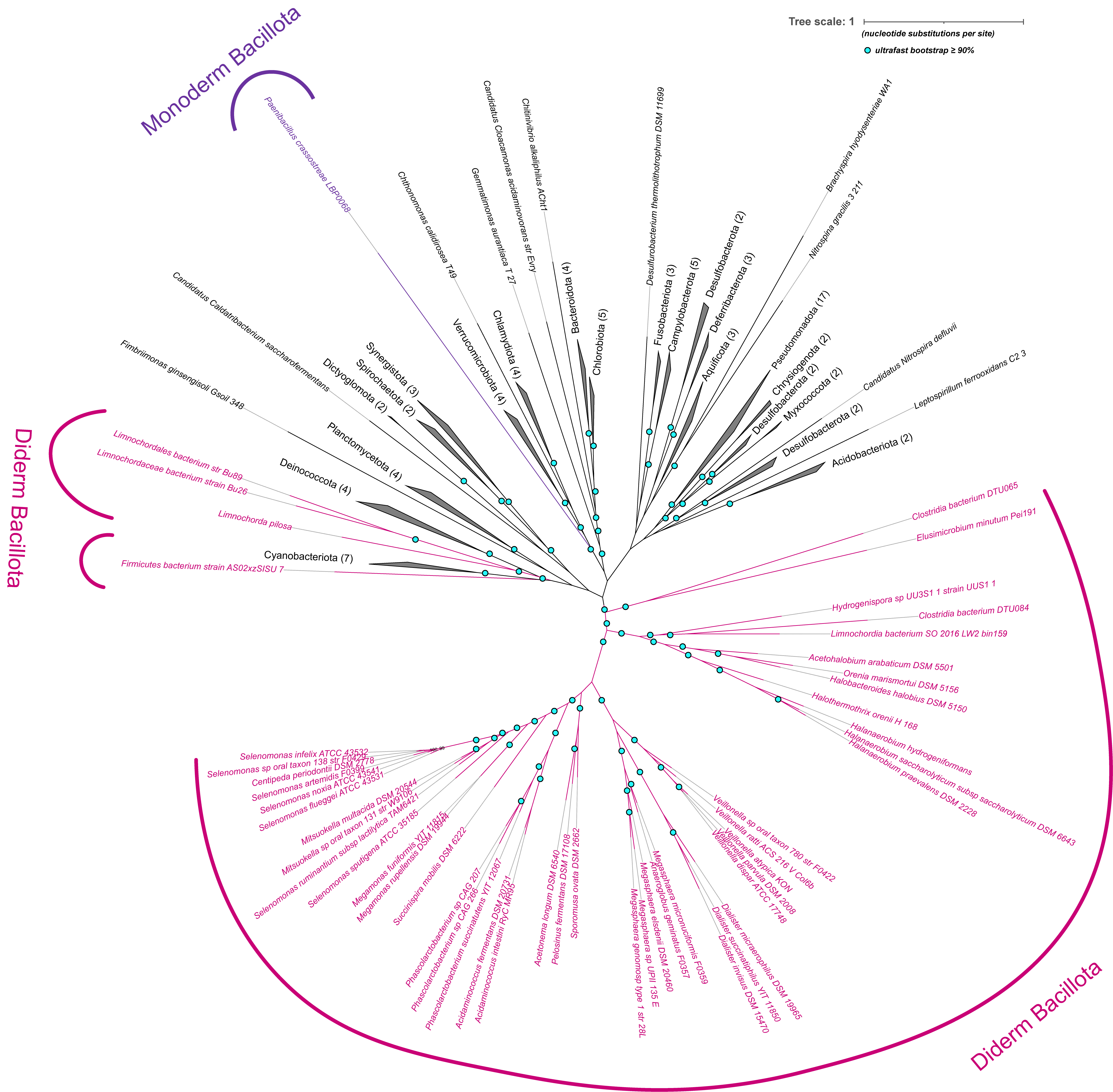

**Figure S1.** **Concatenated LpxADC protein tree.** The concatenated alignments were used as the input to build a maximum-likelihood tree with 3000 ultrafast bootstrap replicates. Branch support greater than or equal to 90% is indicated by blue circles on the branches.


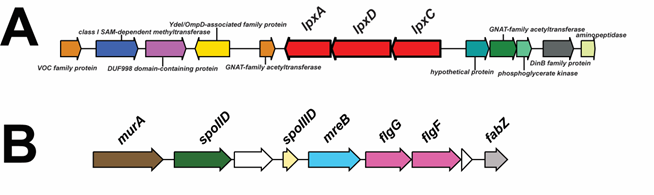


**Figure S2. *Paenibacillus crassostreae LPS-OM genomic regions* (A)**

*lpxACD* region in *Paenibacillus crassostreae* (B) The “scar” LPS-OM biosynthesis cluster region is also present in *P. crassostreae.*


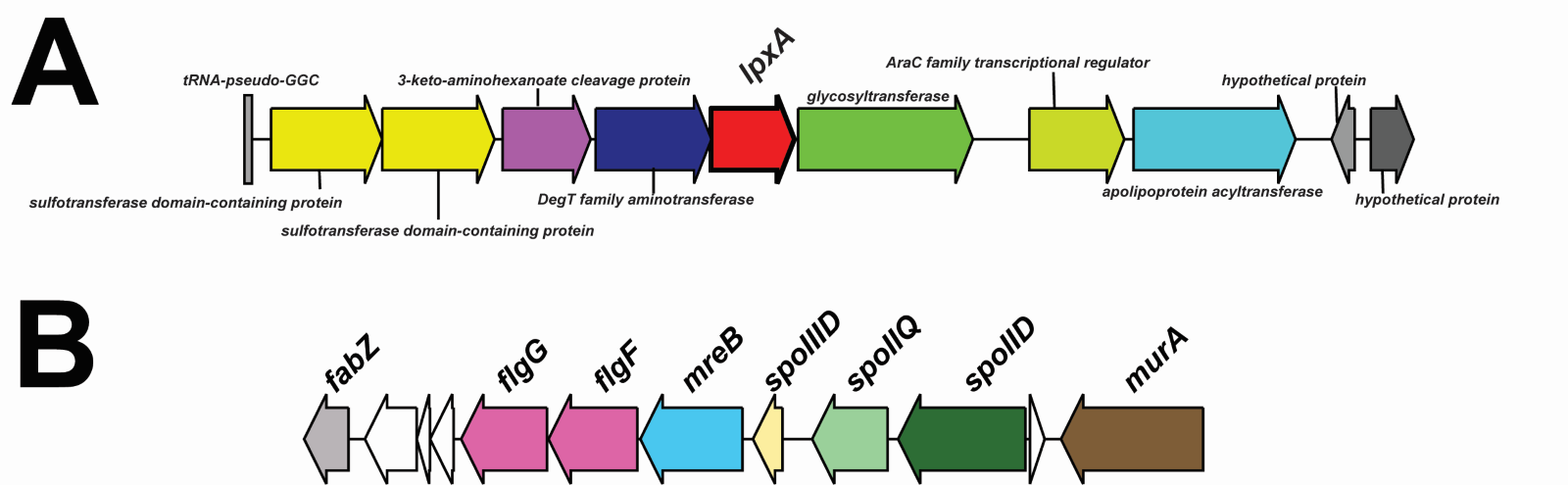


**Figure S3. *Cohnella abietis LPS-OM genomic regions* (A)** LpxA region in *Cohnella abietis*. (B) The “scar” LPS-OM biosynthesis cluster region is also present in *C. abietis.*

**
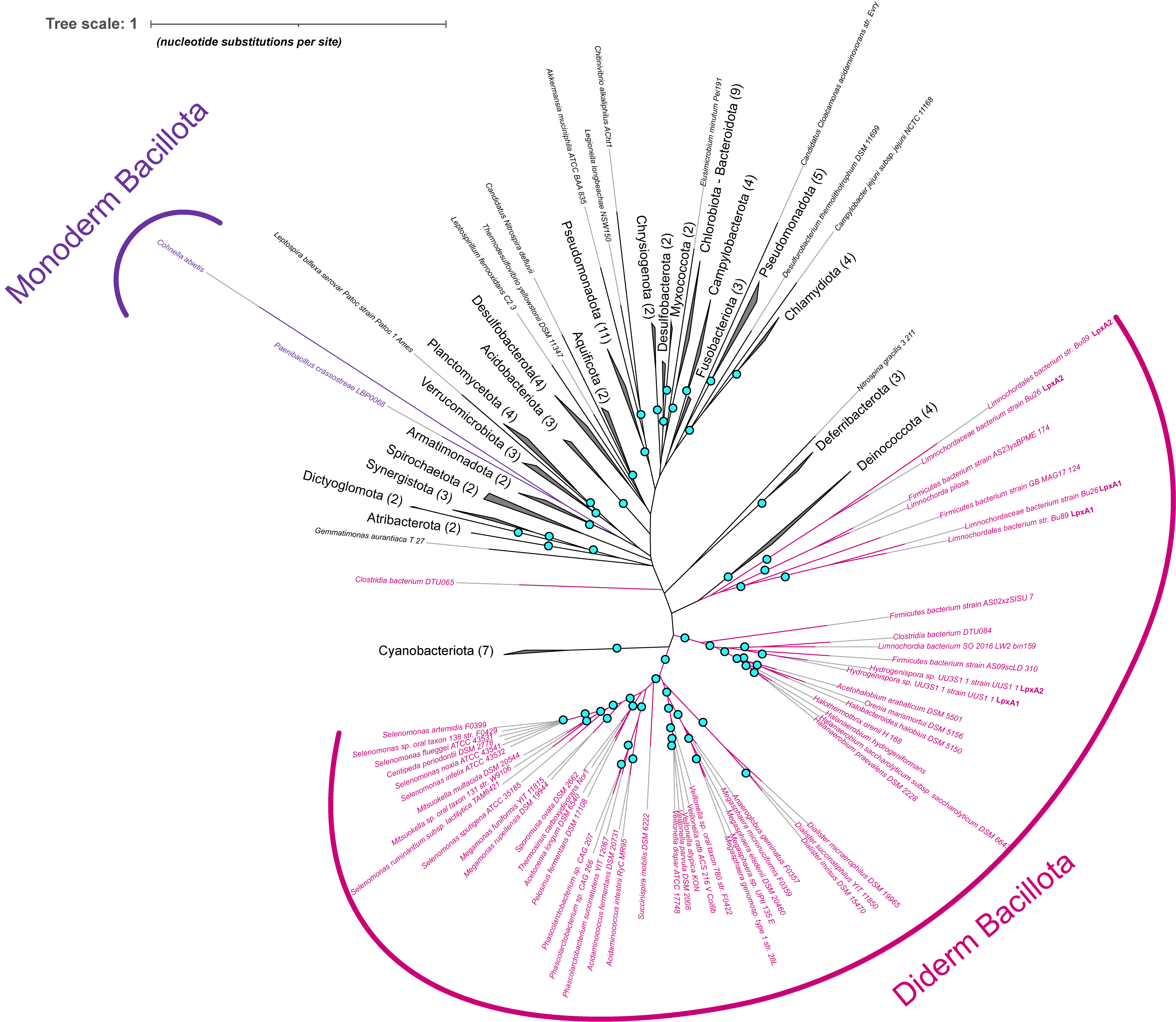
**

**Figure S4.** **LpxA protein tree.** LpxA alignments were used as the input to build a maximum-likelihood tree with 3000 ultrafast bootstrap replicates. Branch support greater than or equal to 90% is indicated by blue circles on the branches.


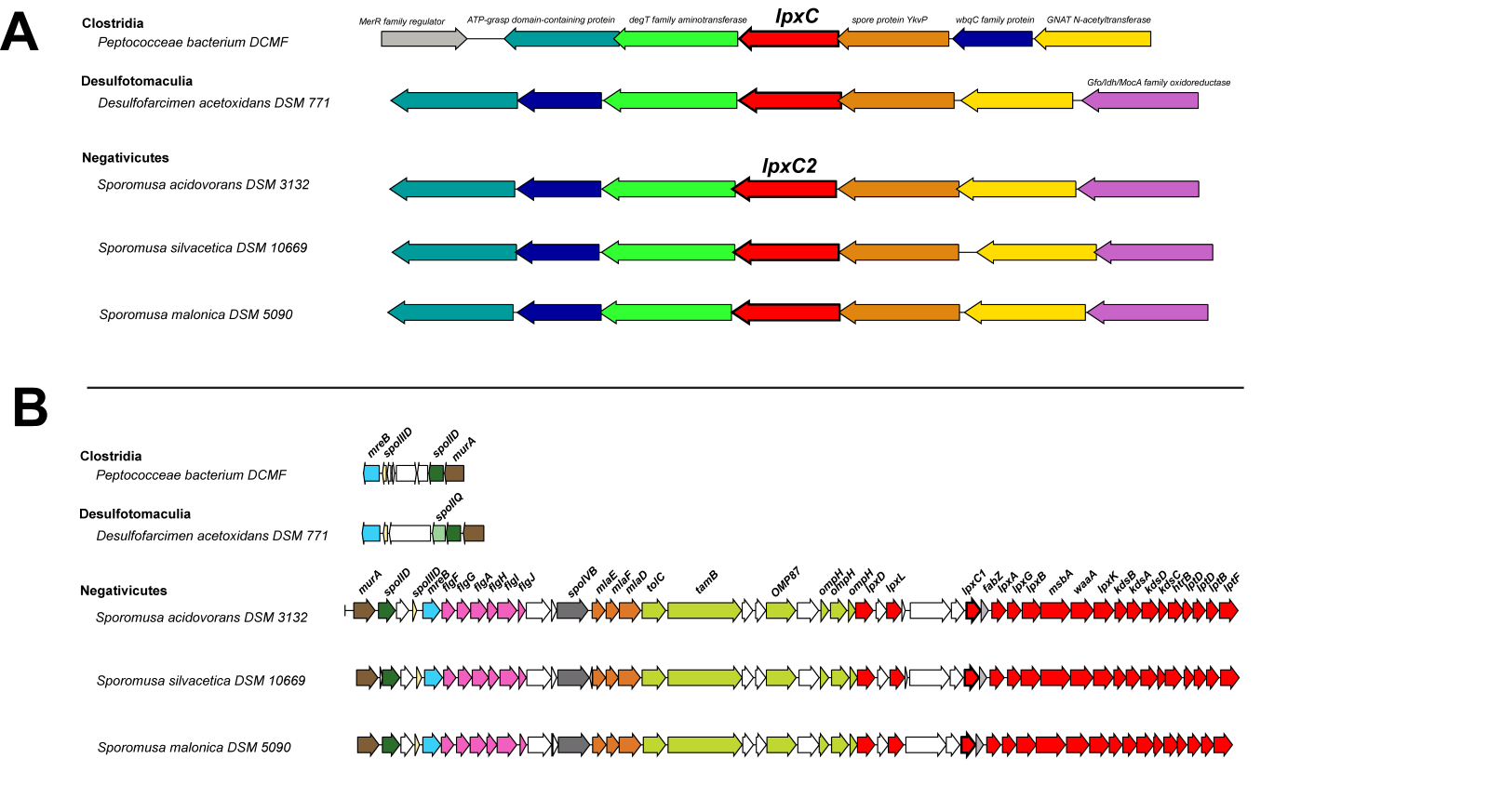


**Figure S5.** **LpxC regions in select monoderm and diderm *Bacillota* strains.** (A) The genomic *lpxC* remnant region that is present in two monoderm lineages within the Clostridia and *Desulfomaculia* and the syntenic *lpxC2* loci that are found in some *Sporomusa* strains. (B) The canonical LPS-OMP biosynthesis gene clusters containing the *lpxC1* gene in the *Negativicutes* strains. A “scar” region is also present in the two monoderm genomes.


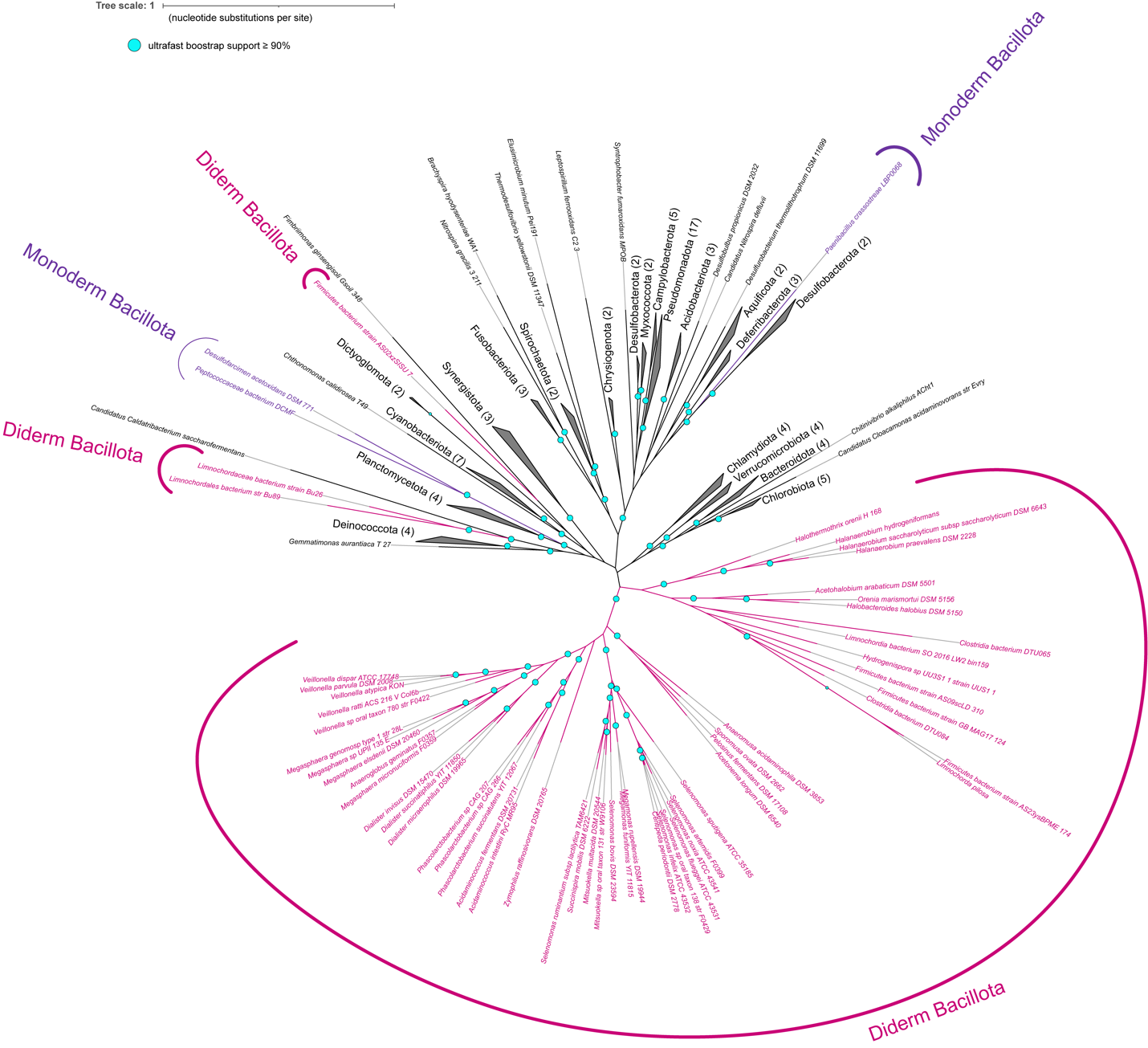


**Figure S6.** **LpxC protein tree.** LpxC alignments were used as the input to build a maximum-likelihood tree with 3000 ultrafast bootstrap replicates. Branch support greater than or equal to 90% is indicated by blue circles on the branches.

**REFERENCES**

1. Simpson BW, Douglass MV, Trent MS. 2020. Restoring Balance to the Outer Membrane: YejM’s Role in LPS Regulation. mBio 11:10.1128/mbio.02624-20.

2. Sperandeo P, Martorana AM, Polissi A. 2019. The Lpt ABC transporter for lipopolysaccharide export to the cell surface. Res Microbiol 170:366-373.

3. Ni D, Wang Y, Yang X, Zhou H, Hou X, Cao B, Lu Z, Zhao X, Yang K, Huang Y. 2014. Structural and functional analysis of the β‐barrel domain of BamA from Escherichia coli. The FASEB Journal 28:2677-2685.

4. Luo Y, Glisson JR, Jackwood MW, Hancock R, Bains M, Cheng I, Wang C. 1997. Cloning and characterization of the major outer membrane protein gene (ompH) of Pasteurella multocida X-73. J Bacteriol 179:7856-7864.

5. Knirel YA, Valvano MA. 2011. Bacterial lipopolysaccharides: structure, chemical synthesis, biogenesis and interaction with host cells. Springer Science & Business Media.

6. Poppleton DI, Duchateau M, Hourdel V, Matondo M, Flechsler J, Klingl A, Beloin C, Gribaldo S. 2017. Outer membrane proteome of Veillonella parvula: a diderm firmicute of the human microbiome. Front Microbiol 8:1215.

7. Eddy S. 1992. HMMER user’s guide. Department of Genetics, Washington University School of Medicine 2:13.

8. Finn RD, Mistry J, Tate J, Coggill P, Heger A, Pollington JE, Gavin OL, Gunasekaran P, Ceric G, Forslund K, Holm L, Sonnhammer ELL, Eddy SR, Bateman A. 2009. The Pfam protein families database. Nucleic Acids Res 38:D211-D222.

9. Taib N, Megrian D, Witwinowski J, Adam P, Poppleton D, Borrel G, Beloin C, Gribaldo S. 2020. Genome-wide analysis of the Firmicutes illuminates the diderm/monoderm transition. Nature Ecology & Evolution 4:1661-1672.

10. Chen SS, Williamson JR. 2013. Characterization of the ribosome biogenesis landscape in E. coli using quantitative mass spectrometry. J Mol Biol 425:767-779.

11. Li H, Handsaker B, Wysoker A, Fennell T, Ruan J, Homer N, Marth G, Abecasis G, Durbin R, Subgroup GPDP. 2009. The sequence alignment/map format and SAMtools. Bioinformatics 25:2078-2079.

12. Sievers F, Higgins DG. 2018. Clustal Omega for making accurate alignments of many protein sequences. Protein Sci 27:135-145.

13. Minh BQ, Schmidt HA, Chernomor O, Schrempf D, Woodhams MD, von Haeseler A, Lanfear R. 2020. IQ-TREE 2: New Models and Efficient Methods for Phylogenetic Inference in the Genomic Era. Molecular Biology and Evolution 37:1530-1534.

14. Kalyaanamoorthy S, Minh BQ, Wong TKF, von Haeseler A, Jermiin LS. 2017. ModelFinder: fast model selection for accurate phylogenetic estimates. Nature Methods 14:587-589.

15. Hoang DT, Chernomor O, Von Haeseler A, Minh BQ, Vinh LS. 2018. UFBoot2: improving the ultrafast bootstrap approximation. Mol Biol Evol 35:518-522.

16. Letunic I, Bork P. 2021. Interactive Tree Of Life (iTOL) v5: an online tool for phylogenetic tree display and annotation. Nucleic Acids Res 49:W293-W296.

17. Beveridge TJ. 1990. Mechanism of gram variability in select bacteria. J Bacteriol 172:1609-1620.

18. Antunes LCS, Poppleton D, Klingl A, Criscuolo A, Dupuy B, Brochier-Armanet C, Beloin C, Gribaldo S. 2016. Phylogenomic analysis supports the ancestral presence of LPS-outer membranes in the Firmicutes. eLife 5:e14589.

19. Capella-Gutiérrez S, Silla-Martínez JM, Gabaldón T. 2009. trimAl: a tool for automated alignment trimming in large-scale phylogenetic analyses. Bioinformatics 25:1972-1973.

20. Coleman GA, Davín AA, Mahendrarajah TA, Szánthó LL, Spang A, Hugenholtz P, Szöllősi GJ, Williams TA. 2021. A rooted phylogeny resolves early bacterial evolution. Science 372:eabe0511.

21. Jiang L, Pheng S, Lee KC, Kang SW, Jeong JC, Kim CY, Park HC, Kim D-H, Kim SW, Kim S-G, Lee J. 2019. Cohnella abietis sp. nov., isolated from Korean fir (Abies koreana) rhizospheric soil of Halla mountain. J Microbiol 57:953-958.

22. Anonymous. *Electrically conductive bacterial nanowires produced by Shewanella oneidensis strain MR-1 and other microorganisms*.

23. Shin S-K, Kim E, Yi H. 2018. Paenibacillus crassostreae sp. nov., isolated from the Pacific oyster Crassostrea gigas. Int J Syst Evol Microbiol 68:58-63.

24. Holland SI, Ertan H, Montgomery K, Manefield MJ, Lee M. 2021. Novel dichloromethane-fermenting bacteria in the Peptococcaceae family. ISME J 15:1709-1721.

25. Widdel F, Pfennig N. 1977. A new anaerobic, sporing, acetate-oxidizing, sulfate-reducing bacterium, Desulfotomaculum (emend.) acetoxidans. Arch Microbiol 112:119-122.

26. Watanabe M, Kojima H, Fukui M. 2013. Desulfotomaculumintricatum sp. nov., a sulfate reducer isolated from freshwater lake sediment. Int J Syst Evol Microbiol 63:3574-3578.

27. Albers U, Tiaden A, Spirig T, Al Alam D, Goyert SM, Gangloff SC, Hilbi H. 2007. Expression of Legionella pneumophila paralogous lipid A biosynthesis genes under different growth conditions. Microbiology 153:3817-3829.
